# A Novel Autophagy-Related Marker for Improved Differential Diagnosis of Rheumatoid Arthritis and Osteoarthritis

**DOI:** 10.3389/fgene.2021.743560

**Published:** 2021-10-12

**Authors:** Rong-zhi Huang, Jie Zheng, Feng-ling Liu, Qing-ling Li, Wen-hui Huang, Dan-meng Zhang, Qiang-chu Wu

**Affiliations:** ^1^ Traumatic Orthopaedic Hand Surgery, The First People’s Hospital of Qinzhou, Qinzhou, China; ^2^ First Clinical Medical School, Guangxi Medical University, Nanning, China

**Keywords:** rheumatoid arthritis, osteoarthritis, identified marker, autophagy, immune

## Abstract

Rheumatoid arthritis (RA) and osteoarthritis (OA) are two most common rheumatic diseases in the world. Although there are standard methods for the diagnosis of both RA and OA, the differentials in some cases are poor. With deepening research, the role of autophagy in maintaining cell homeostasis and thus enabling cells adapt to external environments has become increasingly prominent. Both RA and OA, two diseases with inherent differences in pathogenesis, gradually show differences in autophagy levels. Our study therefore aims to further understand differences in pathogenesis of RA and OA through in-depth studies of autophagy in RA and OA. We also define appropriate autophagy-related markers as recognition indicators. Differences in autophagy levels between RA and OA were found based on analysis of the Kyoto Encyclopedia of Genes and Genomes (KEGG) and single-sample gene set enrichment (ssGSEA). These differences were mainly caused by 134 differentially expressed genes (DEGs). In two autophagy-related genes, *CXCR4* and *SERPINA1*, there existed significant statistical difference between RA and OA. An autophagy related index (ARI) was thus successfully constructed based on *CXCR4* and *SERPINA* by binary logistic regression of the generalized linear regression (GLR) algorithm. Pearson analysis indicated that the expression of *CXCR4*, *SERPINA1*, and ARI were closely correlated with autophagy scores and immune infiltration. Moreover, ARI showed high disease identification through receiver operating characteristic (ROC) analysis (AUC_testing cohort_ = 0.956, AUC_training cohort_ = 0.867). These results were then verified in GSE12021 independent cohort. In conclusion, ARI associated with autophagy and immune infiltration was successfully constructed for accurately identifying OA and RA. The index, thus, has great potential in clinical applications.

## Introduction

With aging population, musculoskeletal-related chronic disability that is caused by both rheumatoid arthritis (RA) and osteoarthritis (OA) can be further aggravated and this imposes huge economic and social burden (Woolf and Pfleger, 2003). Recent epidemiological data showed that from 0.24 to 1% of the people in the world suffered from RA, while OA affected approximately one third of the population over 65 years (Gibofsky, 2014; Johnson and Hunter, 2014). These two are considered the most common rheumatic diseases in the world (Sangha, 2000). A deep understanding of the diseases reveals that although RA and OA have great differences in pathophysiology, similar and overlapping features are presented in their underlying mechanisms (de Lange-Brokaar et al., 2012; Frangos and Maret, 2020). Standard differential diagnoses for RA and OA in some cases lead to poor outcomes (Toes and van der Woude, 2011; Kiltz et al., 2017). There is, thus, an urgent need to further improve their differential diagnosis for better clinical decision-making and early intervention.

Autophagy is a highly conserved intracellular lysosomal-dependent catabolism pathway. It is characterized by the ability of autophagosomes to degrade damaged organelles and maintain cellular homeostasis, thus enabling cell adaption to environmental stress (Mizushima and Komatsu, 2011). Recently, a large number of research showed that autophagy is involved in the development of a variety of rheumatic diseases, including RA and OA (Rockel and Kapoor, 2016; ysl et al., 2016; Marta et al., 2018). In RA, synovial hyperplasia, cartilage degeneration, fibroblast-like synoviocytes (FLS) infiltration into cartilage and bone surfaces, and other pathological features are presented (Pope, 2002). Zhu et al. (2017) showed that autophagy in synovial tissue was significantly enhanced in RA patients, and the level reflected the disease severity. Meanwhile, Shin et al. (2010) indicated that the increased autophagy in RA-FLS promoted RA-associated synovitis because of the decreased rate of apoptosis in RA-FLS-increased synovial fibrosis. On the other hand, the main pathogenesis of OA is the irreversible destruction of cartilage and increase of proinflammatory factors and catabolic enzymes, both of which accelerate the progression of inflammation (Kraan and Berg, 2008). Sasaki et al. (2012) proved that increased autophagy is an adaptive response of cells from stress, this protective mechanism inhibits the expression of genes related to cartilage rupture by regulating apoptosis and reactive oxygen species (ROS), thus, affecting the progress of OA. In addition, Gang et al. (1038) found that increased autophagy could protect chondrocytes against additional apoptosis and senescence. In summary, certain differences in the level of autophagy between RA and OA have been found through these studies, providing hope for the establishment of promising markers of differential diagnosis.

In this study, a comprehensive analysis was used to evaluate autophagy differences between RA and OA. The aim was to establish the importance of autophagy from the perspectives of pathogenesis and diagnosis. Results from this study offer a deeper understanding of autophagy in both diseases and provide assistance for clinical differential diagnosis.

## Materials and Methods

### Data Download and Pre-processing

First, raw data of OA and RA were downloaded from the Gene Expression Omnibus (GEO, http://www.ncbi.nlm.nih.gov/geo/) using GEOquery R package. Next, background correction and normalization with RMA algorithm were processed through the “affy” R package. Probe IDs were transformed to gene symbols by using corresponding R packages (Table 1). Finally, to improve accuracy of analysis, sva algorithm was performed to merge differential datasets and remove batch effect.

**TABLE 1:** The detailed information of the Gene Expression Omnibus (GEO) datasets.

GEO dataset	Platform	Annotation	Sample size
GSE55457	GPL96 (HG-U133A)	hgu133a.db	10 OA and 13 RA
GSE55584	GPL96 (HG-U133A)	hgu133a.db	6 OA and 10 RA
GSE55235	GPL96 (HG-U133A)	hgu133a.db	10 OA and 10 RA
GSE12021	GPL96 (HG-U133A)	hgu133a.db	10 OA and 12 RA

### Identification of Differentially Expressed Genes of Osteoarthritis Versus Rheumatoid Arthritis

GSE55457, GSE55584, and GSE55235 were merged to screen differentially expressed genes (DEGs) between OA and RA through the limma R package. The genes with *p* < 0.05 and |log2FC| > 1 were identified as DEGs.

### Analysis of the Kyoto Encyclopedia of Genes and Genomes and Single-Sample Gene Set Enrichment

In order to explore differences of functional enrichment in OA and RA, the DEGs were implemented by Kyoto Encyclopedia of Genes and Genomes (KEGG) analysis. This was completed with the enrichKEGG function of the clusterProfiler R package. The parameters of the enrichKGG function were used as default (Yu et al., 2012). Autophagy score of each sample was thus calculated by single-sample gene set enrichment analysis (ssGSEA) (Barbie et al., 2009; Hnzelmann et al., 2013). Gene sets for ssGSEA were downloaded from the Human Autophagy Database (HADb, http://www.autophagy.lu/index.html). Differential autophagy levels were identified by Wilcoxon test, with *p* < 0.05 considered as significant statistical difference.

### Acquisition and Verification of Autophagy Related Identified Markers

Overlapped genes among autophagy-related gene set and DEGs were selected as autophagy-related identified markers. Furthermore, an autophagy-related index (ARI) was constructed as identifier model using binary logistic regression of the generalized linear regression (GLR) algorithm. Finally, the GSE12021 dataset was used as a testing cohort to verify differential expression of markers and the accuracy of prediction by ARI.

### Exploration of Immune Microenvironments by Single-Sample Gene Set Enrichment Analysis in Both Osteoarthritis and Rheumatoid Arthritis

RA is a chronic systemic disease mediated by both inflammatory and autoimmune responses (Smolen et al., 2016), while OA is traditionally classified as non-inflammatory arthritis, and predominantly geriatric and degenerative (Glyn-Jones et al., 2015). In recent years, a large number of research studies have also shown that OA is related to individual immunity, but there is still great difference in the levels of immunity between RA and OA (Haseeb and Haqqi, 2013). Hence, we performed ssGSEA analysis to score immune factors for each of the RA and OA samples, based on 29 immune gene sets. Meanwhile, principal components analysis (PCA) was performed to explore differences in immune microenvironments between RA and OA based on scores of the 29 immune factors. The Wilcoxon test was further used to assess potential differences in immune cell environments of RA and OA. A *p* < 0.05 was considered as significant statistical difference.

### Statistical Analysis

Statistical analyses were performed using R (version 4.0.4) (http://www.r-project.org/) and its corresponding packages. The relationship between ARI and the 29 immune factors was determined through correlation analysis. The area under the curve (AUC) of the ROC curve was calculated using the ROCR R package. *p*-Values less than 0.05 indicated statistically significant differences in the analysis.

## Results

### Differentially Expressed Genes of Osteoarthritis Vs. Rheumatoid Arthritis

**FIGURE 1:**
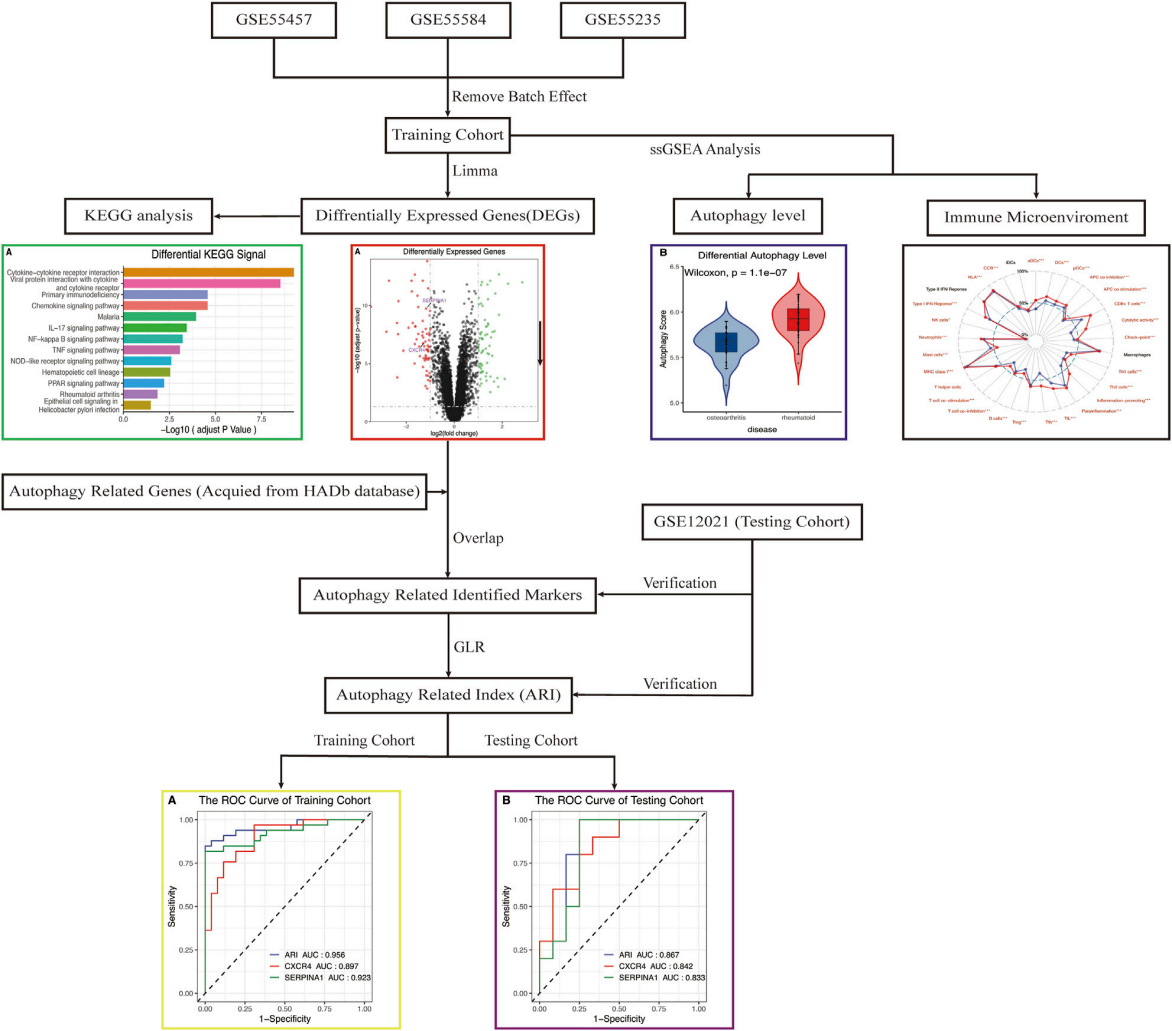
Flowchart of the analysis. DEGs, differentially expressed genes; KEGG, Kyoto Encyclopedia of Genes and Genomes; ssGSEA, single-sample gene-set enrichment analysis; ARI, autophagy-related index; GLR, generalized linear regression; ROC, receiver operating characteristic.

**FIGURE 2:**
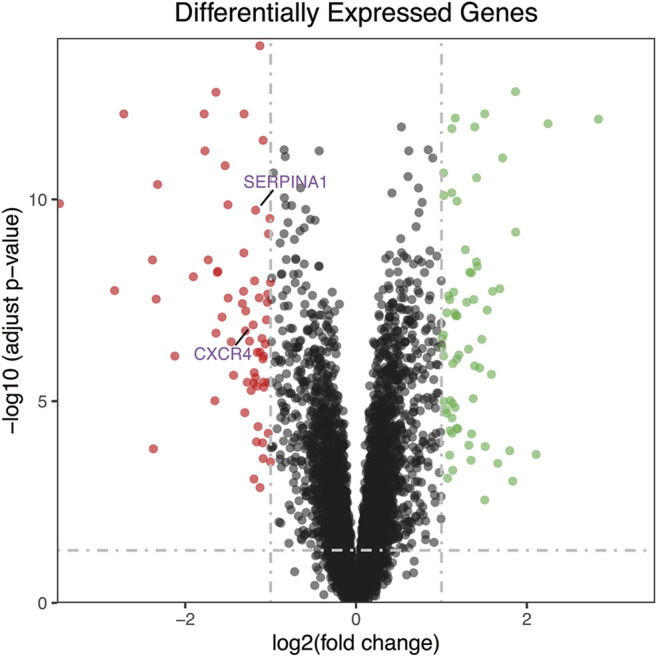
The volcano plot of differentially expressed genes. Red dot represents upregulated genes from comparing OA with RA, while green dot represents downregulated genes. The number of differential encoded genes for both upregulated and downregulated genes was 67. RA, rheumatoid arthritis; OA, osteoarthritis.

The research flow chart is shown in Figure 1. In our study, 134 genes with *p* < 0.05 and |log2FC| > 1 were identified as differentially expressed genes between OA and RA by differential expression analysis. The number of differentially encoded genes for both upregulated and downregulated genes was 67 (Figure 2).

### Kyoto Encyclopedia of Genes and Genomes and Single-Sample Gene Set Enrichment Analyses

**FIGURE 3:**
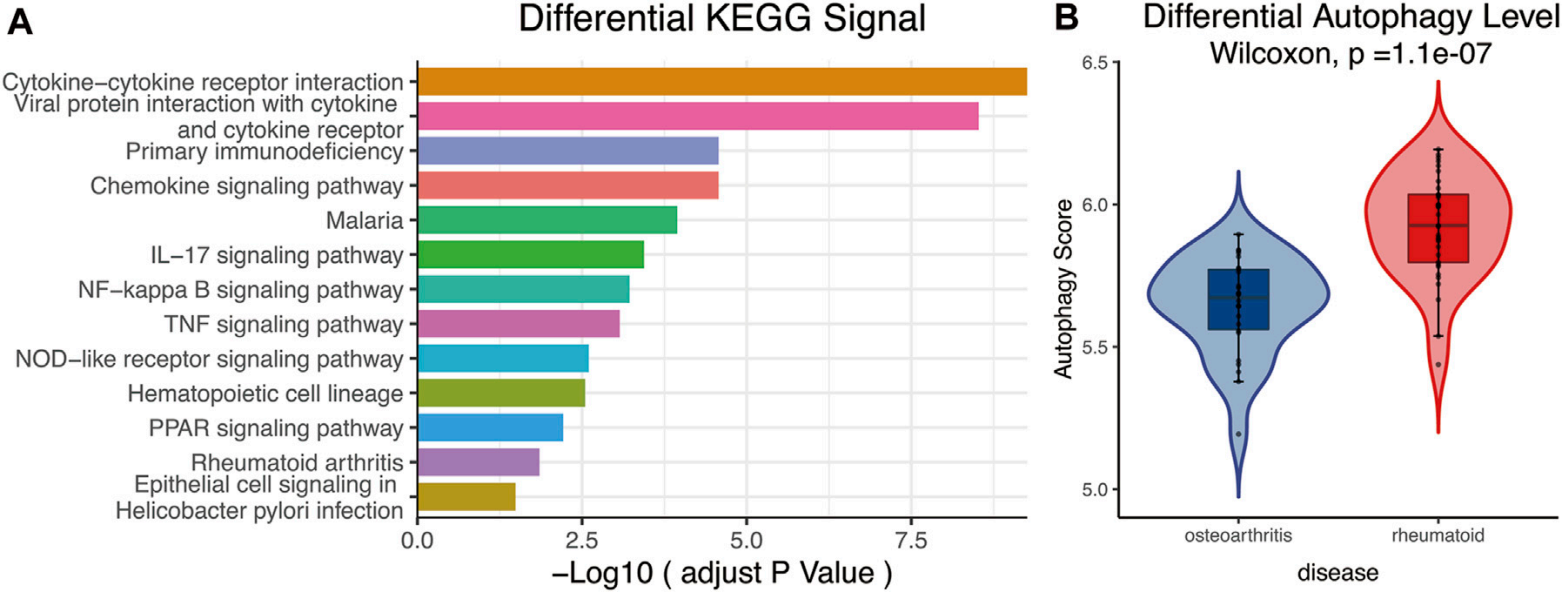
**(A)** KEGG analysis. The different signaling pathways were differentially enriched in RA and OA, and multiple important autophagy-related signaling pathways were present, including NF-kappa B, TNF, NOD-like receptor, and PPAR. **(B)** ssGSEA analysis. Results showed autophagy levels between OA and RA were significantly different, the autophagy level of RA being higher than that of OA. KEGG, Kyoto Encyclopedia of Genes and Genomes; ssGSEA, single-sample gene-set enrichment analysis.

KEGG analysis declared that multiple important autophagy-related signaling pathways were differentially enriched between RA and OA, such as NF-kappa B, TNF, NOD-like receptor, and PPAR (Figure 3A, p_adjust_ < 0.05). These signaling pathways were closely associated with autophagy (Jiang et al., 1998; Lin et al., 2013; Choi et al., 2019; Xu et al., 2019). Moreover, the ssGSEA algorithm showed that significant statistical differences existed between the autophagy levels of RA and OA (Figure 3B, *p* = 1.1e−07). In summary, these analyses indicated that differences in autophagy status are common in the two diseases, RA and OA.

### Acquisition and Verification of Autophagy-Related Identified Markers

**FIGURE 4:**
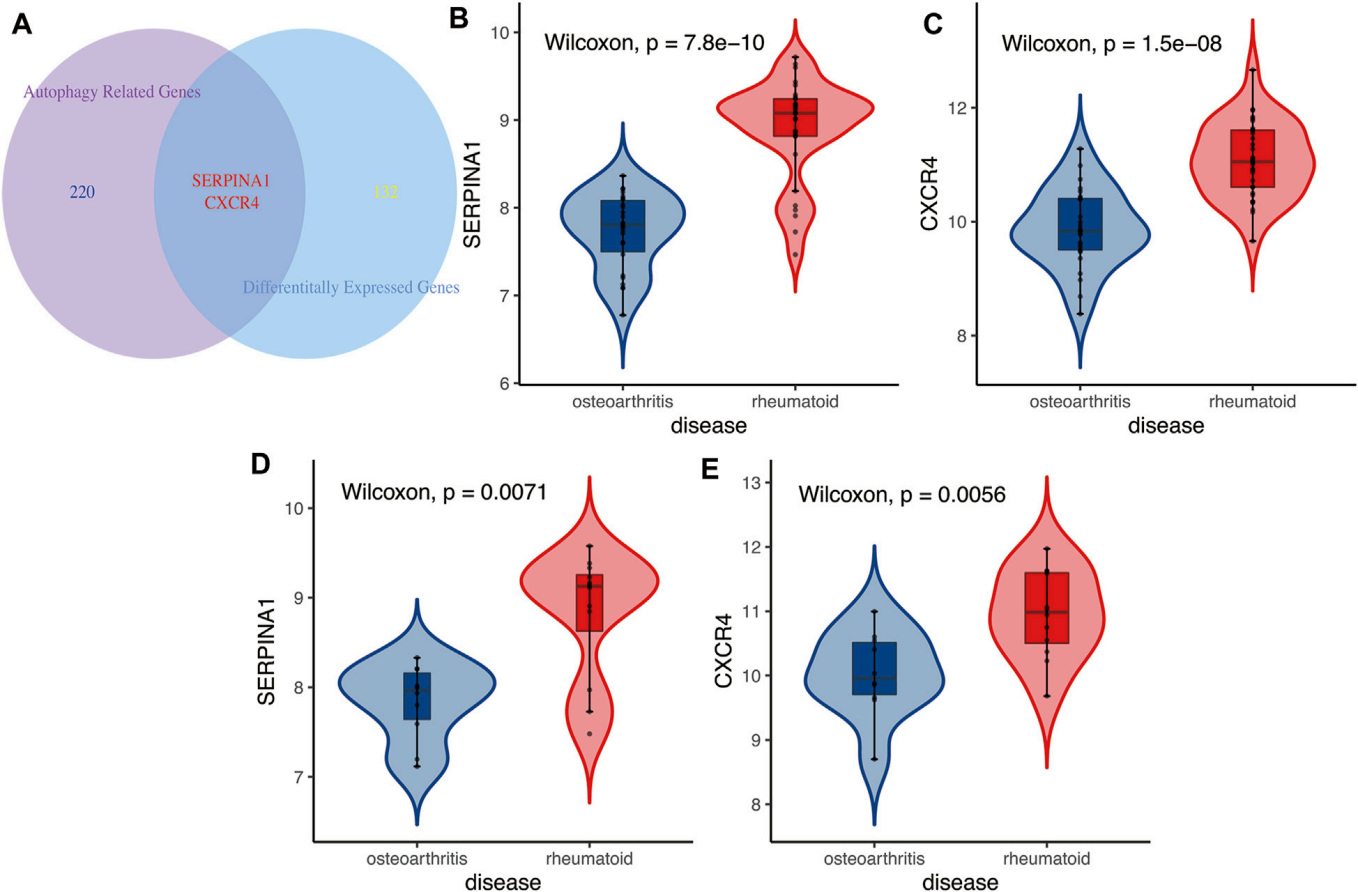
(**A**) The Venn diagram between differentially expressed genes and autophagy-related genes. Results showed autophagy-related genes *CXCR4* and *SERPINA1* were differentially expressed in RA and OA. **(B,C)** Training cohort. Autophagy-related genes *CXCR4* and *SERPINA1* were higher expressed in RA than in OA. **(D,E)** Testing cohort. Results of differential expressions of autophagy-related genes *CXCR4* and *SERPINA1* also verified in validation cohort, higher expressed in RA than in OA.

*CXCR4* and *SERPINA1* associated with autophagy were differentially expressed between OA and RA (Figures 4A–C, *p* < 0.05). The difference was also verified in a testing cohort (Figure 4D,E, *p* < 0.05). Based on these two genes, an ARI was successfully constructed as an identified marker, based on binary logistic regression of GLR:

 logit(p)=ARI=45.941−3.093×ExpSERPINA1−1.968×ExpCXCR4

**FIGURE 5:**
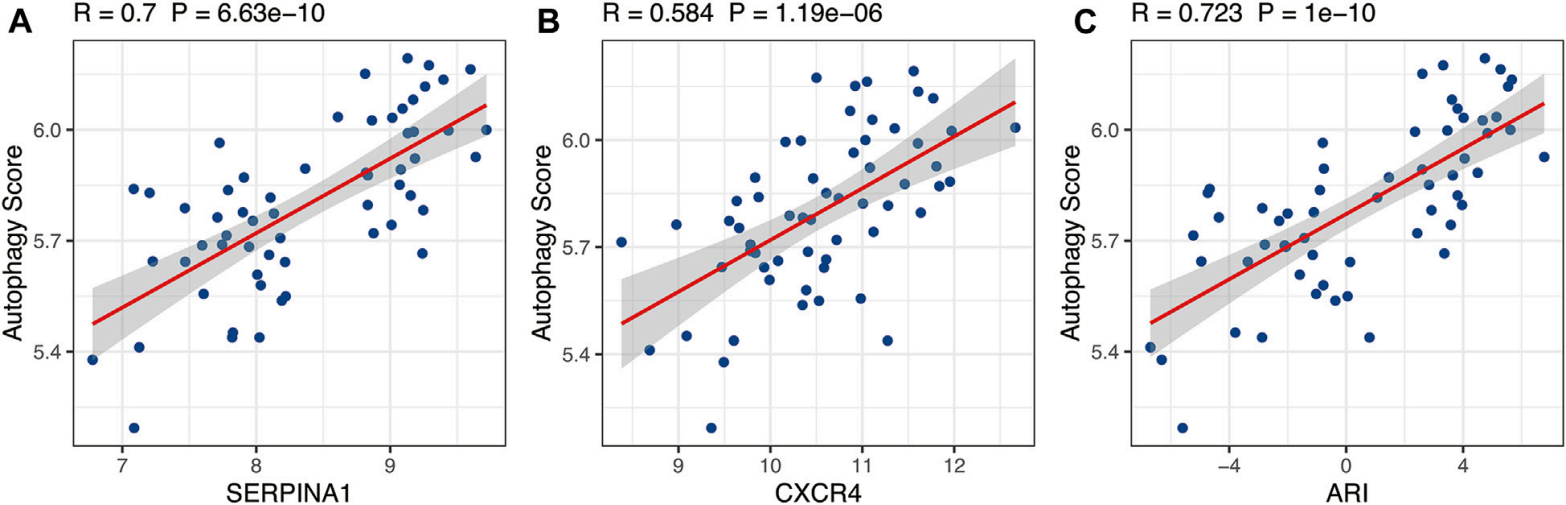
The relationship between autophagy score and *SERPINA1*, *CXCR4*, and ARI. *SERPINA1*, *CXCR4*, and ARI were positively correlated with autophagy level based on Pearson correlation analysis. ARI, autophagy-related index.

Exp_*SERPINA1*_ and Exp_*CXCR4*_ represented the expressions of *SERPINA1* and *CXCR4* genes, respectively, in each sample. The p is the probability of being diagnosed as RA, and 1-p is the probability of being diagnosed as OA. The Pearson correlation analysis demonstrated that ARI was closely positively correlated with autophagy level, an indication that ARI could represent autophagy level (Figure 5C, R = 0.732, *p* = 1e−10). Moreover, the expressions of *SERPINA1* and *CXCR4* genes were also closely positively correlated with autophagy level and was found consistent with the ARI results (Figure 5A, R = 0.7, *p* = 6.63e−10; Figure 5B, R = 0.584, *p* = 1.19e−06).

**FIGURE 6:**
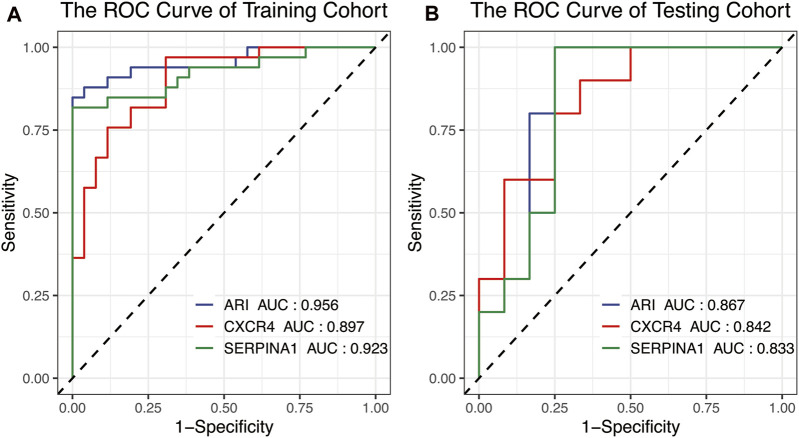
The ROC curve used for the identification accuracy of autophagy-related genes *CXCR4* and *SERPINA1* and ARI for RA and OA. *SERPINA1*, *CXCR4*, and ARI all performed at high accuracy in the **(A)** training cohort and **(B)** testing cohort. ROC, receiver operating characteristic.

To confirm the accuracy and potential ability of differential diagnosis of ARI, ROC values of two cohorts were calculated. Results showed *CXCR4* and *SERPINA1* had high accuracy in the training (Figure 6A, AUC_*CXCR4*_ = 0.897, AUC_*SERPINA1*_ = 0.923) and testing (Figure 6B, AUC_*CXCR4*_ = 0.842, AUC_*SERPINA1*_ = 0.833) sets, and the ARI built from *CXCR4* and *SERPINA1* performed with higher accuracy (Figure 6A, AUC_ARI_ = 0.956, Figure 6B, AUC_ARI_ = 0.867).

### Differential Immune Microenvironments in Rheumatoid Arthritis and Osteoarthritis

**FIGURE 7:**
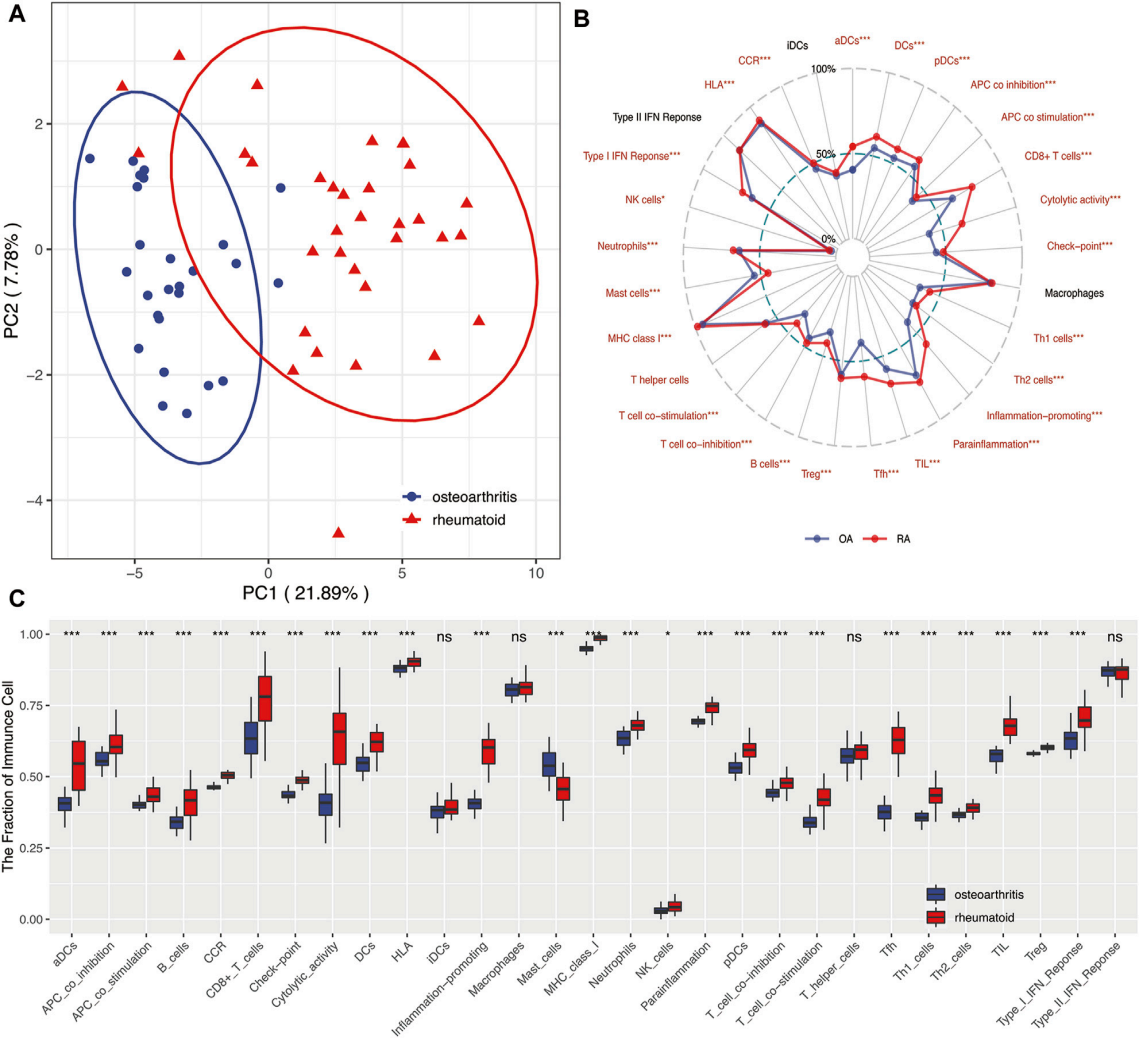
**(A)** PCA showed 29 immune-related factors could distinguish RA from OA to a large extent and indicated RA and OA existed in differential immune environments. **(B)** Radar plot. Summary of scores of 29 immune-related factors between RA and OA. Results indicated that infiltration scores of most immune factors in RA were significantly higher than those in OA. **(C)** Boxplots. Summary of scores of 29 immune-related factors between RA and OA. *means *p* < 0.05, **means *p* < 0.01, and ***means *p* < 0.001. ns, not statistically significant; PCA, principal component analysis.

Figure 7 revealed that OA and RA were significantly divided into two groups based on the scores of 29 immune factors in the PCA analysis. This indicated RA and OA existed with difference in immune environments (Figure 7A). Results of further analysis found that the infiltration score of most immune cells in OA was significantly lower than that in RA, while the infiltration score of mast cells was contrary (Figure 7 B,C, *p* < 0.05).

### Relationship Between Immunity and Autophagy-Related Index

**FIGURE 8:**
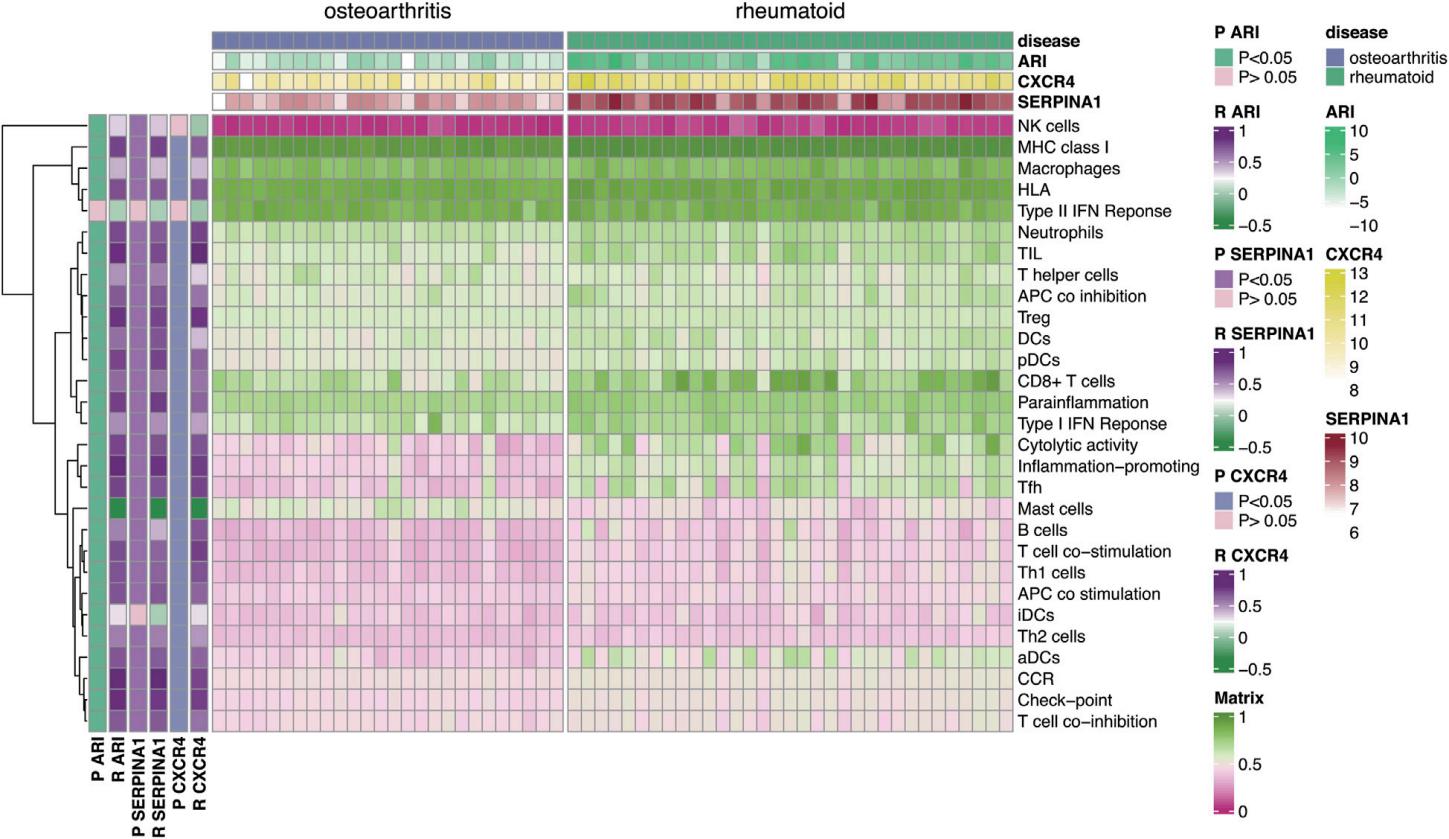
Correlation analysis of autophagy-related genes *CXCR4, SERPINA1*, and ARI with 29 immune-related factors. Results indicated that autophagy-related genes *CXCR4, SERPINA1*, and ARI were positively correlated with most immune-related factors but negatively correlated with mast cells.

To explore relationship between autophagy and immune microenvironments, Pearson correlation analysis was used. Results showed that *CXCR4*, *SERPINA1*, and ARI were positively correlated with most immune factors but negatively correlated with mast cells (Figure 8, *p* < 0.05). This indicated autophagy was closely associated with immune microenvironments in both diseases.

## Discussion

RA is a chronic systemic disease mediated by both inflammatory and autoimmune responses (Smolen et al., 2016; Update on the Pathomechan, 2020; Page et al., 2021). OA which is traditionally classified as non-inflammatory arthritis has predominance as geriatric degenerative disease (Glyn-Jones et al., 2015). In recent years, a deepening understanding of these two diseases, RA and OA, showed similar and overlapping characteristics in their underlying mechanisms (Frangos and Maret, 2020). Therefore, although there are standard differential diagnostic methods for RA and OA, poor differential diagnosis still occurs in some cases (Toes and van der Woude, 2011; Kiltz et al., 2017). Prior research has shown that rheumatoid factor serves as an important index of diagnosis for rheumatoid diseases, with sensitivity and specificity being 60–90% and 85%, respectively. This finding indicates that the diagnosis of some patients with joint pain might not be accurate, especially at the early stages (Mun et al., 2021). Our in-depth review of OA cases found the proportion of such patients was still on the increase (Mobasheri, 2012).

Some studies have shown that many signaling pathways were differentially enriched between OA and RA (Charles, 2017; Tateiwa et al., 2019). In our analysis, we found that diverse differential signaling pathways were mainly mediated by 134 factors. From these, factors like NF-kappa B, TNF, NOD-like receptor, and PPAR have been confirmed to be closely related to autophagy of OA and RA. NF-kB, an important regulator of inflammation and immune response (Dell’Accio and Sherwood, 2015), is involved in many biological processes and its dysregulation is often observed in many conditions like arthritis and autoimmune diseases (Courtois and Gilmore, 2006; Herrington et al., 2015). Choi et al. concluded that targeted intervention in the NF-kB signaling pathway could be used as a treatment method for OA (Choi and Park, 2019). Jiang et al. further demonstrated that dihydroartemisinin could inhibit the NF-kB signaling pathway and thus activate autophagy, inhibiting levels of catabolism and inflammatory factors in the chondrocytes of rats with osteoarthritis (Jiang et al., 2016). TNFα plays a key role in the pathogenesis of RA and it contributes to synovial inflammation and bone degradation (Kobayashi et al., 2000). Lin et al. demonstrated that autophagy was activated in RA in a TNFα-dependent manner and stimulated osteoclast differentiation, thus, promoting the progress of RA (Lin et al., 2013). This affirmed the importance of TNF signaling pathway in RA. Meanwhile, NOD-like receptor signaling pathway was also shown to play an important role in OA. The inflammatory corpuscles, in particular NOD-like receptor protein 3 (NLRP3), are the core of inflammation and a major cause of OA (Zhang et al., 2018). Zhou et al. demonstrated that autophagy could promote the production of IL-1B and inhibit the degradation of mitochondrial components, thus negatively regulating the NLRP3 signaling pathway and preventing its overactivation (Zhou et al., 2011). In addition, Xu et al. showed that the activation of autophagy reduced the activity of NLRP3 in articular cartilage and delayed its degeneration (Xu et al., 2019). In recent years, PPAR was also found to be closely related to energy metabolism, cell differentiation, apoptosis, inflammatory response, and autophagy (Jiang et al., 1998). Studies have shown that PPARγ-mTOR axis played an important role in the pathogenesis of OA which was closely related to autophagy (Dell’Accio and Sherwood, 2015; Vasheghani et al., 2015). These differentially enriched signaling pathways demonstrate the differences between RA and OA in autophagy levels. The importance of autophagy in OA and RA is thus clearly identified by previous studies and ours, and provides basis for further research.

Hence, we believe that autophagy-related genes could identify and reflect differences between these two diseases, RA and OA. Our further analysis found that *CXCR4* and *SERPINA1* played a main role in differential autophagy levels of the two diseases. Relevant studies have shown that *CXCR4* activated autophagy by mediating autophagy signaling pathways, and increased the levels of autophagy proteins and autophagosomes (Hashimoto et al., 2008; Wang et al., 2020). Xia et al. found that low-intensity pulsed ultrasound activated autophagy and enhanced the SDF-1/CXCR4 signaling pathway, thereby promoting the migration of mesenchymal stem cells and contributing to the enhancement of cartilage repair effect on OA (Xia et al., 2021). This further suggests that *CXCR4* is of significance as an autophagy-related identification marker of OA and RA. Although the role of *SERPINA1* in autophagy has not been studied much in OA and RA, it has been shown to be associated with autophagy in a variety of other diseases (Teckman et al., 2004; Jxd et al., 2020). A likely role of *SERPINA1* in RA and OA could thus be explored. An ARI composed of *CXCR4* and *SERPINA1* and established by us, served as identified markers of RA and OA. The accuracy of the ARI was verified in an independent cohort. The ARI should have great potential in clinical applications.

Rheumatoid arthritis (RA) is believed to be significantly related to autoimmunity, and immunotherapy is one current treatment method for the disease (Firestein and McInnes, 2017). In recent years, greater attention has been paid to the relationship between immunity and osteoarthritis (OA), and the role of immunity in the pathogenesis of OA was considered important (Rollín et al., 2008; Martel-Pelletier et al., 2016). However, the role of immunity in the two diseases was still different (Haseeb and Haqqi, 2013; Smolen et al., 2018). Our analysis gave results consistent with those of previous studies. The infiltration score of most immune-related factors in RA was significantly higher than that in OA (de Lange-Brokaar et al., 2012). Notable also was that the infiltration scores of mast cells were higher in OA than in RA. This was consistent with most immunological studies on OA (Dean et al., 1993; Pu et al., 1998), but some studies still reported lower numbers of mast cells (MCs) in OA compared with RA (Gotis-Graham and Mcneil, 2014). Moreover, the specific mechanism of the influence of mast cells on OA and RA was still not fully understood (Rivellese et al., 2017). The association of mast cells with local inflammation, chondrocyte apoptosis, cartilage breakdown, and positive autoantibodies (Rivellese et al., 2019; Wang et al., 2019) is cause for further study. We could thus confirm that immunity played an indispensable role in RA and OA, and further research on it ought to provide new directions for improving the diagnosis and treatment of RA and OA in the future.

Interestingly, our analysis found that the ARI was positively correlated with a variety of immune cells, while negatively correlated with mast cells. It could be inferred that autophagy was related to immunity in RA and OA, this being also consistent with results from a large number of studies on autophagy and immunity. Autophagy is closely related to immune functions such as intracellular bacterial clearance, inflammatory cytokine secretion, lymphocyte development, and pro-inflammatory signal transduction (Levine et al., 2011). Relevant studies have proved that autophagy-related gene polymorphisms were associated with a variety of autoimmune diseases, including rheumatoid arthritis, systemic lupus erythematosus, psoriasis, and multiple sclerosis (Wu and Adamopoulos, 2017). Masaru et al. proved that autophagy was associated with the survival of RA fibroblastic synovial cells (RA-FLs), and Ra-FLs are the primary source of pro-inflammatory cytokines responsible for the activation of osteoclasts and subsequent bone destruction (Kato et al., 2014). Wang et al. indicated that monocyte chemoattractant-protein-induced protein 1 (MCPIP-1) could upregulate autophagy-related protein Beclin-1 and microtubule-associated protein 1A/1B-light chain 3 (LC3) in monocytes, thereby promoting an initial differentiation of monocytes into osteoclasts by increasing endoplasmic reticulum stress and oxidative stress, which was closely related to bone resorption in OA (Wang et al., 1093). Thus, autophagy was closely related to immunity in RA and OA, and any correlation between ARI and immunity would further enhance the feasibility and potential of our identification indicators.

In general, the ARI could reflect not only the differences in autophagy but also the differences in immunity in OA and RA. This further increases the potential ability and reliability of ARI in differentiating RA and OA. The ARI composed of two genes was expected to provide new ideas for the diagnosis and treatment of OA and RA and further research on new therapeutic drugs. However, there are some limitations to the study that are worth considering. We included independent datasets for validation but this study was retrospective as the number of datasets was small. Therefore, a well-designed prospective study is needed in the future to further confirm the discriminative ability and accuracy of the ARI.

## Conclusion

In this study, a comprehensive autophagy-related index (ARI) consisting of autophagy-related genes *CXCR4* and *SERPINA1* was successfully constructed for distinguishing RA and OA. The ARI showed a high differential diagnostic accuracy and could well reflect the immune and autophagy status of RA and OA. To a certain extent, this provides hope for the difficulties in distinguishing RA from OA. This finding is conducive to prompt implementation of clinical decision-making and intervention measures.

## Data Availability

Publicly available datasets were analyzed in this study. This data can be found here: http://www.ncbi.nlm.nih.gov/geo/) GSE55457, GSE55584, GSE55235, GSE12021.

## Abbreviations

ACPAs, anti-citrulline protein antibodies; ARGs, autophagy-related genes; ARI, autophagy-related index; AUC, area under the curve; DEGs, differentially expressed genes; FLS, fibroblast-like synoviocytes; GEO, Gene Expression Omnibus; GLR, generalized linear regression; HADb, Human Autophagy Database; KEGG, Kyoto Encyclopedia of Genes and Genomes; NLRP3, NOD-like receptor protein 3; OA, osteoarthritis; PCA, principal components analysis; RA, rheumatoid arthritis; RF, rheumatoid factor; ROC, receiver operating characteristic; ROS, reactive oxygen species; ssGSEA, single-sample gene-set enrichment analysis
